# The optimal dose of ribavirin for chronic hepatitis C: From literature evidence to clinical practice: The optimal dose of ribavirin for chronic hepatitis C

**Published:** 2011-04-01

**Authors:** Ludovico Abenavoli, Marta Mazza, Piero L. Almasio

**Affiliations:** 1 Department of Experimental and Clinical Medicine, University Magna Gracia of Catanzaro, Catanzaro, Italy; 2 Gastrointestinal and Liver Units, DI.BI.M.I.S, Policlinico, University of Palermo, Palermo, Italy

**Keywords:** Hepatitis C virus, Ribavirin, dose-response

## Abstract

Approximately 170 million people worldwide are chronically infected by hepatitis C virus (HCV), which can result in progressive hepatic injury and fibrosis, culminating in cirrhosis and end-stage liver disease. The benchmark therapy for untreated HCV patients is a combination of pegylated interferon-alpha (PEG-IFN) and ribavirin (RBV). Several studies have suggested several potential new approaches to improve HCV therapy-optimization of the dose and duration of RBV therapy, accompanied by careful clinical management, is crucial in ensuring the greatest likelihood of a long response to therapy. RBV causes serious side effects, but in clinical practice, there are no alternatives for the treatment of HCV infection. Based on our results, weight-based doses of RBV are advantageous for genotype 1-infected patients, but its success in genotype 2- and 3-infected patients is unknown, particularly for shorter treatment durations.

## Background

Approximately 170 million people worldwide are chronically infected by hepatitis C virus (HCV) [1], which can result in progressive hepatic injury and fibrosis, culminating in cirrhosis and end-stage liver disease. Among adults in the Western world, chronic hepatitis C (CHC) is the major cause of cirrhosis and the principal indication for liver transplantation. CHC also contributes to the increasing incidence of hepatocellular carcinoma (HCC), for which few satisfactory therapies exist [2]. The primary treatment goal in patients with chronic HCV infection is viral eradication. The benchmark therapy for untreated HCV-patients is a combination of pegylated interferon-alpha (PEG-IFN) and ribavirin (RBV) [3]. HCV genotype should be systematically determined before treatment, because it dictates the indication, treatment duration, RBV dose, and virological monitoring procedure [4]. HCV genotype 2- and 3-infected patients require 24 weeks of treatment and a low dose of RBV-i.e., 800 mfg daily.

In contrast, HCV genotype 1-, 4-, 5-, and 6- infected patients require 48 weeks of treatment and a higher, body weight-based dose of RBV-i.e., 1000-1400 mg daily [4]. This combination therapy is highly successful in patients infected with genotypes 2 and 3, effecting a sustained virologic response (SVR)-defined as undetectable serum HCV RNA by quantitative PCR 24 weeks after the end of treatment-ranging between of 76% and 82% [5][6]. There is strong evidence that a treatment duration of 24 weeks yields equivalent SVR rates as 48 weeks [7]. However, SVR rates in patients with genotype 1 infections, which constitute approximately 70% of cases of CHC in the USA [8], are lower, wherein 42% to 46% of patients achieve SVR after 48 weeks of combination therapy. Several new, potent HCV protease and polymerase inhibitors have been described recently [9], but none is available in clinical practice. Higher response rates are observed in the majority of patients who are able to tolerate and adhere to RBV, suggesting that cumulative RBV exposure is important. Optimization of RBV dose and duration of therapy, in conjunction with careful clinical management, is crucial in ensuring the greatest chance for a durable response to the therapy.

This report will review the clinical role of RBV and, in particular, the selection and maintenance of the optimal RBV dosing strategy that are required to achieve sustained viral suppression in patients with chronic HCV infection.

## Current treatment schedule

Combination therapy with PEG-IFN and RBV has been reported in large clinical trials to effect high SVR rates and, correspondingly, low rates of virologic relapse [10]. However, the response rate to antiviral therapy varies according to HCV genotype. HCV genotypes 2 and 3 are more responsive to therapy than genotype 1, having comparatively higher SVR rates with most therapeutic options [11][12]. Despite the good response of genotype 2 and 3 patients to therapy, there is still a clear benefit of adding RBV to therapy with PEG-IFN, and SVR rates of approximately 80% have been reported with this combination [13]. The impact of PEG-IFN and RBV on the response of other HCV genotypes [4][5][6] has not been as well examined, because these genotypes are rarer and tend to be pooled in analyses or excluded altogether from larger trials. Although patients with genotype 1 infection are generally less responsive to therapy, an SVR to combination therapy is still observed in approximately 50% of such patients [5][14]. A large, randomized, controlled study, comparing PEG-IFN alpha-2a alone (180 µg/week) with PEG-IFN alpha-2a plus RBV (1000/1200 mg/day) or interferon alpha-2b (3 MU thrice weekly) plus RBV over 48 weeks clearly demonstrated that RBV significantly improves outcomes in genotype 1-infected patients [6].

## Ribavirin in the treatment of HCV chronic infection

RBV monotherapy is not efficacious against chronic HCV infection. Some placebo-controlled clinical trials have shown that RBV reduces serum transaminase levels and HCV RNA concentrations, but both parameters returned to pre-treatment levels after the therapy was halted [15][16]. Moreover, RBV alone had no effects on liver histology. When it is combined with standard or PEG-IFN, RBV enhances the virological, biochemical, and histological response compared with IFN alone [12][17]. Further development of this therapeutic model, taking into account the anti-HCV activity of RBV, has fit well with the experimental data, showing that the addition of RBV enhances SVR rates by approximately 25% to 30% and suggesting a mechanism by which RBV enhances declines in HCV RNA and improves long-term outcome [18].

Reductions in RBV dose negatively affect SVRs in patients who are infected with HCV genotype 1, and higher RBV doses are associated with higher SVR rates in patients who are treated with PEG-IFN alpha-2b plus RBV [19]. In addition, genotype 1-infected patients who were randomized to PEG-IFN alpha-2a plus 1000/1200 mg/day RBV had higher SVR rates than those given PEG-IFN alpha-2a plus 800 mg/day RBV, further indicating the significance of adequate RBV dosing [5]. Both the timing of dose reduction and lower relative doses affect SVR. For example, a retrospective analysis showed that reductions in RBV dose during the first 12 weeks of treatment affected the early virologic response (EVR), defined as undetectable HCV RNA < 50 IU/mL by qualitative PCR or a ≥ 2-log decrease in HCV RNA at Week 12 in patients infected with HCV genotype 1 [20].

## Assessment of ribavirin dose

**Figure 1:**
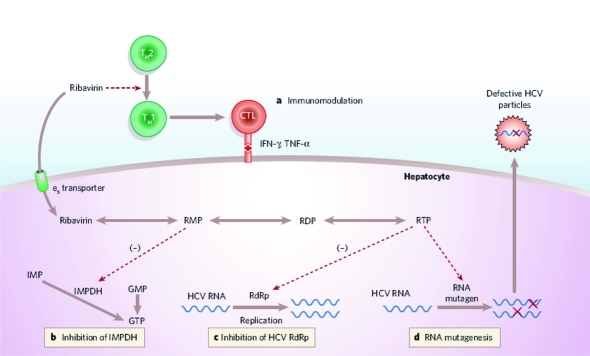
Proposed mechanisms of action of ribavirin. IMPDH, inosine monophosphate dehydrogenase; TH, T helper cell. TNF, tumor necrosis factor. (Reprinted with permission from J.J. Feld and J.H. Hoofnagle. Mechanism of action of interferon and ribavirin in treatment of hepatitis C Jordan J. Nature 2005, 436:967-972.)

A physician who prescribes RBV must select the appropriate starting dose, maintaining it by careful clinical management of any RBV-related side effects and incrementally decreasing the dose, and he must determine when the patient should return to the indicated dose following successful management of side effects. It is also crucial to continue RBV to the end of therapy if possible, because late discontinuation can adversely affect clinical outcomes. RBV monotherapy does not induce a significant antiviral response in patients with chronic HCV infection, but in combination with IFN, it markedly improves the rate of end of treatment response (ETR)-defined as undetectable serum HCV RNA at the end of the treatment-reduces relapse rate,s and increases SVR rates. Since the synthesis of RBV in the early 1970s, several mechanisms of action have been proposed and might vary between viruses. For the treatment of chronic HCV infection, the following mechanisms are currently considered: (i) immunomodulatory properties, (ii) inhibition of inosine monophosphate dehydrogenase (IMPDH), (iii) direct inhibition of the HCV-encoded NS5B RNA polymerase, (iv) induction of lethal mutagenesis, and (v) modulation of interferon-stimulated gene (ISG) expression [21][22] (Figure 1).

A relationship between RBV dose and response to therapy with both IFN alpha-2a and alpha-2b has been established in genotype 1 patients, who benefit from doses that exceed 800 mg/day [5][14]. When RBV is combined with PEG-IFN alpha-2a, relatively small reductions to 800 mg/day lead to significantly lower rates of SVR [5]. Similarly, a large comparative trial of fixed-dose RBV compared with weight-based dosing in combination with PEG-IFN alpha-2b demonstrated that stratifying patients of all genotypes to receive starting doses ranging from 800-1400 mg/day depending on weight effects higher SVR rates than using a fixed dose of 800 mg/day for all patients [7]. A detailed analysis of the relationship between body weight and SVR has suggested that the dose per kilogram is the determining factor of response in genotype 1 patients, based on the 40% to 50% rise in SVR for a 12-16-mg/kg increase in RBV dose [23]. RBV dose by weight may impact its concentration in plasma, which also correlates with the response. Although this relationship has been well documented in genotype 1 patients, the data are less clear for other genotypes. A relationship between response and plasma concentration has been proposed for non-genotype 1 patients [24]. However Snoeck et al. observed no effect of dose per kilogram body weight on SVR in genotype 2/3 patients [23].

Based on these data, weight-based dosing has been used more extensively in patients with genotype 1 HCV, and it is required to achieve maximum efficacy. The standard initial dose of RBV in patients with HCV genotype 1 is 1000/1200 mg/day (1000 mg/day = 75 kg; 1200 mg/day > 75 kg) over a 48 weeks, although higher RBV doses are considered for patients > 85 kg. A study of 380 patients has shown that the pharmacokinetics of RBV vary widely, wherein lean body weight emerges as the only factor that influences clearance, supporting the use of these two distinct weight-based doses in patients with genotype 1 disease [25]. Although data modeling from patients who received this standard starting dose suggests that SVR increases linearly with RBV doses that equate to > 10 mg/kg, the rate of anemia also rises linearly simultaneously (< 10 g/dL hemoglobin) [23]. An RBV dose of 15 mg/kg/day might achieve the best balance between efficacy and a manageable safety profile.

The impact of dose reduction on SVR was assessed retrospectively by analyzing drug exposure in genotype 1-infected patients who were randomized to PEG-IFN alpha-2a (180 µg/week) plus RBV (1000 or 1200 mg/day) and completed 48 weeks of treatment [4]. Neither EVR nor SVR was adversely affected by reductions in RBV dose, as long as the cumulative ribavirin exposure was greater than 60% of the intended dose, whereas the SVR rate in patients who received a lower dosage of RBV was significantly lower (33% vs. 64%; p < 0.0001). Notably, RBV dose reductions during weeks 5-48 had minimal impact on SVR in patients who achieved rapid virologic response (RVR), defined as undetectable serum HCV RNA levels at 4 weeks, even when the cumulative RBV dose was less than 60% of the intended dose. In patients who did not achieve RVR, however, reductions in RBV dose after Week 4 had a negative impact on SVR rate in all RBV exposure categories.

When non-responders to IFN, with or without RBV, were re-treated with PEG-IFN alpha-2a plus RBV, RBV dose reductions during Weeks 1-20 were associated with reduced a SVR rate (21% to 11%; p = 0.031), whereas later RBV dose reductions did not affect SVR rates. In addition, patients who were initially treated with IFN mono-therapy had a higher probability of attaining a SVR during retreatment with PEG-IFN alpha-2a plus RBV than those who were initially treated with interferon and RBV. Further analysis in re-treated patients has shown that reductions in RBV dose during the first 12 or 20 weeks of treatment do not significantly affect SVR rates as long as patients remain on full-dose PEG-IFN alpha-2a. However, discontinuation of RBV during the first 20 weeks of therapy or drug interruption for at least 7 consecutive days during the first 12 weeks had a negative impact on SVR. In addition, RBV dose reductions during the following 20-40 weeks did not have a consistent effect on SVR, as long as PEG-IFN alpha-2a dose was maintained and RBV was not discontinued [26].

To assess the role of RBV in HCV clearance and evaluate the consequences of RBV discontinuation, 516 patients who were infected with HCV genotype 1 were treated with 180 µg/week of PEG-IFN alpha-2a and 800 mg/day of RBV. Those subjects who were HCV RNA-negative at Week 24 were randomized to further treatment with PEG-IFN alpha-2a plus RBV or PEG-IFN alpha-2a alone [27]. Responders at Week 24 who stopped RBV had higher rates of breakthrough during treatment and relapse after therapy than those who continued on both agents (SVR rates, 52.8% vs 68.2%; p = 0.004). However, the side effect profile and quality of life of patients who discontinued RBV tended to improve. Recently, Ferenci et al. have been investigated efficacy and tolerability of 24 weeks of treatment with RBV 800 mg/day or 400 mg/day plus PEG-IFN alpha-2a 180 µg/week in 141 treatment-naïve patients who were infected HCV genotype 2 or 3. Data suggests that 400 mg/day of RBV enough in patients infected with HCV genotype 3 to achieve as high SVR rates as those attained by the standard 800 mg/day dosing (SVR: 63.9% versus 67.5%), whereas the same results could not be replicated in patients with HCV genotype 2. In the latter patients the SVR rates following low-dose RBV were significantly lower than those attained with a standard dose of RBV (55.6% versus 77.8%) [28].

Recent studies suggest that high-dose RBV in combination with PEG-IFN can improve responses in genotype 1-infected patients. Lindahl et al. used an individualized dosing regimen, based largely on renal function, in an attempt to achieve a steady-state RBV concentration greater than 15 µmol/l in 10 treatment-naïve patients [29]. After initial dose adjustments, the mean dose of RBV was 2,540 mg per day (range 1,600-3,600 mg), and the mean RBV concentration was 14.7 µM (range 7.8-22.0 µM) at Weeks 24-48. Nine of 10 patients achieved an SVR, but the side effects, in particular anemia that required erythropoietin, were much more frequent and severe. The impact of dose reduction on SVR was assessed retrospectively by analyzing drug exposure in genotype 1-infected patients who were randomized to PEG-IFN alpha-2a (180 mg/week) plus RBV (1000 or 1200 mg/day) who completed 48 weeks of treatment [30]. Neither the EVR or SVR was adversely affected by RBV dose reduction as long as cumulative RBV exposure was > 60%, whereas the SVR rate in patients receiving > 60% RBV was significantly lower than in those receiving > 60% RBV (33% vs 64%; p < 0.0001). Patients who received ≤ 60% RBV dosing experienced prolonged periods of dose reduction, interruption of therapy, or premature discontinuation. Even in patients with a > 97% cumulative RBV dose over Weeks 1-12, the SVR rate was significantly lower in those with ≤ 80% RBV exposure than in those with > 80% exposure during Weeks 13-48 (48% vs 67%; p = 0.372). In contrast, RBV dose reductions during Weeks 5-48 had a minimal impact on SVR in patients who achieved RVR, even when the cumulative RBV dose was < 60%. In patients who did not achieve RVR, however, RBV dose reductions after Week 4 had a negative impact on SVR rate in all RBV exposure categories.

More recently, in a prospective, open-label, randomized, controlled pilot study comparing 48 weeks of treatment with PEG-IFN plus standard weight-based RBV with or without erythropoietin (groups 1 and 2) and PEG-IFN plus higher weight-based RBV plus erythropoietin (group 3), SVR was significantly greater in group 3 patients due to a significant decline in relapse rate [31].

## Ribavirin dosage and adverse events

The main serious adverse event that is associated with the use of RBV is dose-dependent hemolytic anemia. Anemia is frequently observed in patients receiving combination treatment with standard interferon or PEG-IFN plus RBV [5][12]. RBV-induced anemia has been shown to be primarily affected by plasma RBV concentration, not by dose per kilogram body weight [32]. A recent publication supports the individualization of RBV dosing according to HCV genotype and body weight and highlights several clinical variables that have an effect on the likelihood of SVR versus the occurrence of anemia [23]. A higher apparent clearance of RBV, older age, and cirrhosis have a negative impact on achieving an SVR. Gender and RBV dose/kg are the most important prognostic factors for the occurrence of anemia. However, because anemia is not a universal risk in all treated patients, the initial high-dose strategy of 1,000 or 1,200 mg per day based on body weight, appears to be appropriate. For heavier patients, RBV doses greater than 1,200 mg/day may be initiated, because they are likely to be associated with additional efficacy and a manageable risk of anemia [23].

Few studies have shown that erythropoietin can be used to improve quality of life, maintain RBV dose, and subsequently improve adherence to therapy (33, 34). Although erythropoietin may have a role in the management of RBV-related anemia, a recent study failed to show an improvement of SVR in genotype 1-infected patients who were given epoetin alpha at the initiation of therapy to maintain hemoglobin levels between 12 and 15 g/dL (35). This was a three-arm, prospective, open-label, randomized, controlled pilot study comparing 48 weeks of treatment with PEG-IFN plus standard weight-based RBV with or without erythropoietin (groups 1 and 2) and PEG-IFN plus higher weight-based RBV plus erythropoietin (group 3). A signiﬁcantly smaller percentage of group 2 patients experienced a decline in hemoglobin to less than 10 g/dL (9% vs 34%; p < 0.05) and required a more frequent dose reduction of RBV compared with group 1 patients (10% vs 40%; p < 0.05). Nevertheless, SVRs were similar in the two latter groups (19% vs. 29%). SVR was signiﬁcantly higher in group 3 patients (49%) due to a relevant decline in relapse rate.It has been suggested that the use of erythropoietin may be an appropriate strategy for managing anemia, improving quality of life, and increasing adherence to therapy, particularly in patients with genotype 1 infection [29]. However, its use has not been permitted in registration trials of PEG-IFNs and RBV, and no recommendation for its use in RBV-associated anemia has been included in EU and USA labels [36][37]. The limited data available concerning the use of erythropoietin are insufficient to make clear recommendations. If shortening the treatment below the standard duration is to be considered, careful reassessment of RBV dose is necessary, because RBV dose and treatment duration appear to be closely linked. In a prospective study, reducing the dose of RBV to 400 mg did not adversely affect the rate of SVR compared with the standard 800 mg daily dose in genotype 2- and 3-infected patients who were treated for 24 weeks [28].

Another adverse event that is associated with RBV is loss of bone mineral density (BMD). When parameters of BMD were assessed in 13 patients who were treated with interferon alone and 19 who were treated with interferon plus RBV, the latter had signi?cantly lower BMD (1.108 ± 0.08 g/cm2 vs. 0.877 ± 0.07 g/cm2; p < 0.001), T-scores (0.19 ± 0.6 vs -1.94 ± 0.6; p < 0.001), and Z-scores (0.26 ± 0.6 vs. -1.7 6 ± 0.5; p < 0.001) by magnetic resonance imaging; urinary calcium excretion (218 ± 97 mg/24 h vs. 76 ± 36 mg/24h; p < 0.001); and calcium/creatinine ratios (1.9 ± 0.3 mg/mg vs. 0.06 ± 0.02 mg/mg; p < 0.01) [38]. Other studies reported no loss in BMD in a group of 12 patients with recurrent HCV infection after orthotopic liver transplantation [39].

Pre- and post-treatment measurements showed no differences in BMD between 13 pediatric patients who were treated with PEG-IFN alpha-2b and RBV and 7 patients who were treated with interferon alone [40]. Although incubation of human osteoclast-like cells with interferon for up to 14 days had no effect on cell growth, RBV significantly reduced cell proliferation, increased cell death, and reduced alkaline phosphatase (ALP) activity, indicating that it suppresses osteoblast differentiation [41][42]. In contrast, RBV had little effect on the proliferation or ALP activity of murine osteoblasts and no direct effect on osteoclast differentiation or function, although it indirectly induced TRANCE/RANKL gene expression in osteoblasts, thus enhancing osteoclast formation [43]. These ?ndings suggest that the involvement of RBV in reducing BMD is unclear, necessitating further study.

Lower doses of RBV may also be appropriate in certain patient groups who can not otherwise tolerate RBV therapy, such as those with renal impairments. With careful monitoring of plasma concentrations to avoid overdosing, RBV doses of 200-800 mg/day have been successfully administered in a small cohort of renally impaired patients [19]. The literature suggests that 200-400 mg/day of RBV can be given safely and may allow for more successful treatment [30]. Recurrence of HCV chronic infection is universal in patients who require liver transplantation for this indication, but many transplant recipients have some degree of renal impairment. In a group of transplant patients who were treated with interferon and RBV, the incidence of hemolysis was related to the degree of renal impairment, suggesting that the RBV dose should be adjusted to reduce hemolysis [44]. In addition, pharmacokinetic studies in HCV-positive kidney or liver transplant patients have shown that RBV dosage is dependent on renal function and that monitoring plasma ribavirin concentrations during treatment can maximize efficacy while reducing side effects [45].

## Discussion

The milestone in contemporary therapy of chronic HCV infection is to deliver doses of both agents that confer optimal antiviral efficacy for a sufficient time to minimize viral relapse. At the same time, it is important to minimize the impact of side effects that might erode the effectiveness of therapy due to dose reductions below the level of therapeutic efficacy or because the patient is unable to complete an optimal treatment course. The association between RBV and PEG-IFN improves SVR rates and decreases the rate of relapse in patients with HCV infection. Thus, combined treatment is considered the benchmark therapy. A number of mechanisms, including direct inhibition of RNA replication, immunomodulation, inhibition of inosine monophosphate dehydrogenase, and enhanced viral mutagenesis, have been proposed to explain the role of RBV.

However, many questions remain open [28][46]. Clinical evidence has shown the importance of RBV in the treatment of hepatitic C infection, and research suggests a number of potential approaches to optimizing HCV therapy to increase SVR rates. However, this drug is associated with frequent side effects, necessitating dose reductions and/or discontinuation. For this reason, patient management is required to monitor the toxicities of therapy; in particular, hemoglobin levels should be monitored in patients with risk factors for treatment-induced hemolytic anemia, and dose reductions or other therapeutic interventions should be administered in a timely manner [47]. Dose reductions in RBV should be limited to the minimum that is required to address side effects, possibly using small decrements and avoiding reductions to below 60% of the target dose whenever possible.

At present, there are no alternatives to RBV for the treatment of HCV infection, and therefore, maintaining patients on their indicated dose and length of therapy is crucial if the goal of a high rate of SVR is to be achieved. However, due to the limited data available, further studies on RBV dose and treatment duration are warranted before any recommendations can be made. In our opinion, weight-based dosing of RBV is advantageous for genotype 1-infected patients, while its relevance for genotype 2- and 3-infected patients remains to be further clarified, particularly for shorter treatment durations.
